# Nationwide Comparison of ICU Procedure Frequencies in Japan Using a Public Open Database: A Cross-Sectional Study by ICU Admission Fee Type and Region

**DOI:** 10.3390/jcm15062341

**Published:** 2026-03-19

**Authors:** Yuko Kawamura, Aiko Tanaka, Osamu Nagata, Yuka Matsuki

**Affiliations:** 1 Department of Intensive Care, University of Fukui Hospital, Fukui 910-1193, Japan; yuko0131@u-fukui.ac.jp (Y.K.); aikotanakaicu@gmail.com (A.T.); 2 Department of Anesthesiology and Intensive Care Medicine, Graduate School of Medicine, Osaka University, 2-15 Yamadaoka, Suita 565-0871, Japan; 3 Department of Anesthesiology and Reanimatology, Faculty of Medical Sciences, University of Fukui, Fukui 910-1193, Japan; o-nagata@fa2.so-net.ne.jp

**Keywords:** intensive care unit, open healthcare data, nationwide analysis, practice patterns

## Abstract

**Background/Objectives:** Publicly available open databases offer advantages in terms of accessibility and transparency. However, their application in intensive care research remains limited. Therefore, in this study, we examined whether simple nationwide comparisons of intensive care unit (ICU) practice patterns are feasible using an open database. **Methods:** A multicenter, cross-sectional study was conducted using data from the Bed Function Report. ICU wards reimbursed under ICU admission fee types were included and classified as high-acuity or standard ICUs. The ward-level procedure frequencies of procedures, including mechanical ventilation, were calculated. Comparisons were performed according to ICU admission fee type and geographic region. Quasi-Poisson regression models with offsets for annual ICU admissions were applied, accounting for overdispersion. **Results:** A total of 602 ICUs were included in the study. Non-metropolitan ICUs demonstrated higher procedural rates for mechanical ventilation compared with metropolitan ICUs (rate ratio [RR], 1.11; 95% confidence interval [CI], 1.02–1.21). Standard ICUs consistently had lower procedural rates for mechanical ventilation than high-acuity ICUs (RR, 0.74; 95% CI, 0.68–0.81). Group analyses indicated that regional differences in procedure frequencies were evident in standard ICUs, but not in high-acuity ICUs. **Conclusions:** This study demonstrated the feasibility of comparing ICU practice patterns across different regions and facility types in Japan using a nationwide open public database. This approach may serve as an initial step in a stepwise research framework that links open-database profiling to patient-level analysis using more detailed data sources.

## 1. Introduction

Nationwide healthcare database research is essential for evaluating healthcare delivery systems at the national level, informing regional healthcare planning, and developing medium- to long-term strategies for workforce allocation and infrastructure investment [1]. Publicly available open databases, in particular, provide accessibility and transparency, and multiple such databases have been developed internationally as valuable research resources [2]. In Japan, the Bed Function Report, mandated annually by the Ministry of Health, Labour, and Welfare for hospitals and clinics with general or long-term care beds, serves as a nationwide open database covering all medical institutions, regardless of size or care structure. These data are publicly accessible via government websites [3]. The database has been used in previous studies for regional comparisons and healthcare system evaluations across diverse clinical and policy contexts, including dental–hospital care coordination, work productivity assessment, and perinatal care system organization [4,5,6]. However, relatively few studies have utilized this database.

Other nationwide studies in Japan often rely on the Diagnosis Procedure Combination (DPC) database or the National Database of Health Insurance Claims and Specific Health Checkups, both of which include data from all medical institutions and are widely used in research [7]. Nevertheless, access to these databases requires formal application procedures and data purchase. By contrast, many nationwide comparative studies on intensive care have relied on specialized clinical registries containing detailed patient-level information. The Japanese Intensive Care Patient Database (JIPAD) is a nationwide registry based on voluntary ICU participation [8]. As continuous data entry is required, participation is skewed toward larger hospitals; as of 2023, the registry included 129 facilities, resulting in limited nationwide coverage and acknowledged selection bias [9,10,11,12]. Although the Bed Function Report contains limited clinical details due to its nature as an open administrative database, its comprehensive nationwide coverage is a major strength. When research questions are appropriately defined, this database may be useful for comparing structural characteristics and practice patterns across healthcare delivery systems.

The ICU is a care setting for critically ill patients requiring continuous or frequent monitoring and organ support, and differences in organizational structure are likely to be reflected in the types and frequencies of procedures performed. In Japan, ICUs are classified into four admission fee types (types 1–4) based on structural differences in staffing and facility requirements. Resource-rich ICUs with greater staffing and equipment availability are associated with lower mortality rates than standard ICUs with limited resources [13]. More broadly, healthcare research in Japan has documented physician concentration in urban areas, chronic shortages in less populated regions [14], and disparities in healthcare access and service provision between urban and non-urban areas [15]. Similar patterns of physician maldistribution have been reported in other healthcare systems [16]. However, comprehensive evaluations of such disparities in intensive care remain limited.

Therefore, in this study, we aimed to examine the feasibility of simple nationwide comparisons using the publicly available Bed Function Report as an open database. As an application example, we conducted nationwide comparisons of procedure frequencies reflecting continuous monitoring and organ support in intensive care according to ICU admission fee type and geographic region.

## 2. Materials and Methods

### 2.1. Study Design and Data Sources

This multicenter cross-sectional study used data from the 2023 Bed Function Report [3], which includes information collected between April 2023 and March 2024. This nationwide report provides ward-level data on the frequency of medical procedures reimbursed by the Japanese Health Insurance System. Data were electronically collected based on ward codes attached to insurance claims; neither the hospital nor ward names were anonymized. Monthly counts of reimbursed medical procedures and inpatient admission fees were recorded, and information on healthcare staff was aggregated at the ward level.

This study used publicly available, de-identified data and was exempt from ethical review in accordance with the regulations of the Research Ethics Committee of Fukui University. Informed consent was not required.

### 2.2. Eligibility Criteria

Wards for which reimbursement claims for ICU admission fees were recorded in the Bed Function Report were identified. When multiple ICU admission fee types were claimed for the same ward during the study period, the type with the highest number of claims was assigned as the representative admission fee type. Wards reimbursed under ICU admission fee types 1 and 2 were classified as high-acuity ICUs, whereas those reimbursed under types 3 or 4 were classified as standard ICUs.

Wards clearly designated as pediatric-only units, based on names indicating “Pediatric Intensive Care Unit (PICU),” were excluded. Additionally, when a medical institution had one ward explicitly labeled as an ICU (e.g., “ICU” or “Intensive Care Unit”) and another ward with a name suggestive of a general ward that claimed ICU admission fees, the latter ward was excluded. This exclusion was necessary because accurately identifying the ward primarily providing intensive care was challenging, and including such wards carried a high risk of misclassification and double-counting.

### 2.3. Variables

Each ICU ward was classified according to the ICU admission fee type as described above [13].

Procedure frequency was estimated based on four representative ICU procedures as proxy indicators of clinical activity in the ICU: arterial line placement (AL), central venous catheter insertion (CVC), mechanical ventilation (MV), and continuous renal replacement therapy (CRRT).

For each ICU ward, the number of patients who underwent each procedure was identified from the monthly data reported in the Bed Function Report and aggregated over 12 months. Similarly, the total number of patients for whom ICU admission fees were claimed during the same 12-month period was calculated and considered the annual number of ICU admissions to that ward. The procedure rate for each ward was defined as the number of patients who underwent a given procedure divided by the number of patients for whom ICU admission fees were claimed. Procedure rates were compared across groups stratified by geographic region and type of ICU admission fee.

$$ICU\;procedure\;rate=\frac{number\;of\;procedure\;(12\;months\;total)}{number\;of\;ICU\;fee\;requests\;(12\;months\;total)}$$

In the Bed Function Report, the procedure counts were masked to protect patient confidentiality when the number of cases was small. Specifically, a value of “0” is displayed when no procedures were performed, whereas counts between 1 and 9 are masked with an asterisk. To address these masked values, a median value of 5 was imputed for the primary analysis. Sensitivity analyses were performed using the minimum (imputed value of 1) and maximum (imputed value of 9) possible values.

The geographic classification of each ICU was based on secondary medical areas, which is commonly used in healthcare system analyses in Japan [14,17]. The ICUs were categorized into three groups according to the location of the affiliated hospital.

- Metropolitan: areas with a population density ≥ 1000 persons/km^2^ or a total population ≥ 1,000,000;
- Suburban: areas with a population density of 200–1000 persons/km^2^ or a total population ≥ 300,000;
- Rural: areas with a population density of <200 persons/km^2^ and a total population < 300,000.

### 2.4. Statistical Analyses

Statistical analyses were conducted in three steps. All analyses were performed using R (version 4.5.0; R Software for Statistical Computing, Vienna, Austria), and a two-sided *p*-value < 0.05 was considered significant.

#### 2.4.1. Descriptive Analysis and Comparison of Categorical Variables

ICU wards were classified according to the ICU admission fee type (high-acuity vs. standard) and geographic category (metropolitan, suburban, or rural). Differences in the distribution of ICU admission fee types across geographic categories were evaluated using Pearson’s chi-square or Fisher’s exact tests, as appropriate. Distributions of procedure rates are summarized as medians with interquartile ranges according to the ICU admission fee type and geographical category.

#### 2.4.2. Primary Analysis: Assessment of Regional Differences Using Poisson Regression

The primary outcome was the procedural rate at the ward level. For each procedure—AL, CVC, MV, and CRRT—Poisson regression models were fitted, with the annual number of procedures per ward as the dependent variable. Geographic category (metropolitan vs. non-metropolitan) and ICU admission fee type (high-acuity vs. standard) were included as explanatory variables, and the annual number of ICU admissions (number of ICU admission fee claims) was incorporated as an offset term.

Overdispersion was assessed using the Pearson chi-square statistic divided by the degrees of freedom (χ^2^/df), with a value > 1.5 indicating overdispersion. Quasi-Poisson models were applied when over-dispersion was present. The results were reported as rate ratios (RRs) with 95% confidence intervals (95% CIs). As the annual number of ICU admissions was included as an offset, the model estimates correspond to relative differences in procedure rates per ICU admission. The rate ratio represents the ratio of procedure rates between comparison groups, adjusted for the annual number of ICU admissions through the offset term. An RR > 1 indicates a higher procedure rate relative to the reference group, whereas an RR < 1 indicates a lower rate. A quasi-Poisson model including an interaction term (geographic category × ICU admission fee type) was compared with a main-effects-only model using an F-test.

#### 2.4.3. Group Analyses

Group analyses were performed by stratifying the data according to the ICU admission fee type (high-acuity vs. standard) and geographic category. The differences in procedure rates by region or ICU admission fee type were evaluated within each stratum.

#### 2.4.4. Use of Generative Artificial Intelligence

In the statistical analyses, generative artificial intelligence (ChatGPT, version 5.1; OpenAI, San Francisco, CA, USA) was used to assist with drafting and refining R code and clarifying statistical concepts. All analytical decisions, interpretation of results, and conclusions were made by the authors. Artificial intelligence was not used to generate data, make statistical modeling decisions, or create tables or figures.

## 3. Results

### 3.1. Characteristics

**Figure 1:**
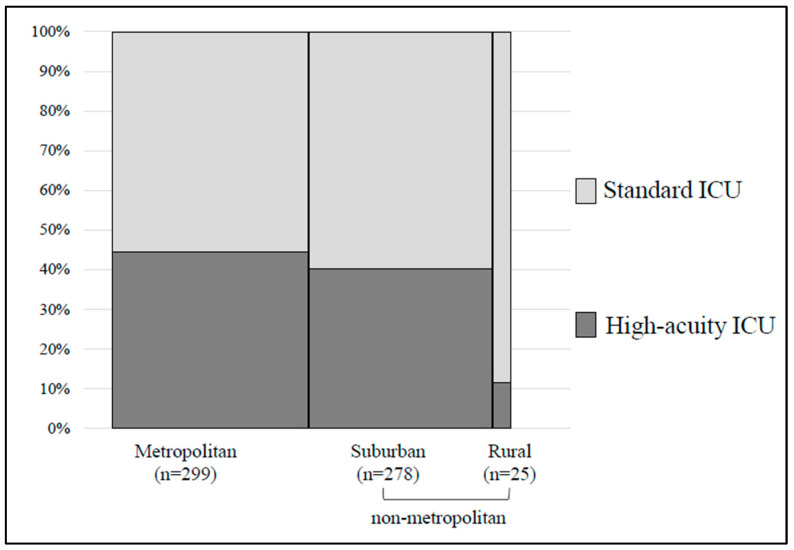
Distribution of intensive care unit (ICU) admission fee types by geographic categories.

From the 2023 Bed Function Report, 625 wards claiming ICU admission fees were identified. Of these, 11 pediatric-only wards labeled as PICUs and 12 wards with names suggestive of general wards were excluded, resulting in a final analytical sample of 602 ICU wards. The distribution of ICU wards by admission fee type, geographic category, and basic regional characteristics, including population size, is summarized in Table 1. In metropolitan areas, population size and the number of physicians were comparable to those in suburban areas; however, because the geographic area was approximately ten times smaller, population density and physician density were roughly ten times higher than in suburban areas. High-acuity ICUs accounted for 44.4% of ICUs in metropolitan areas, representing a higher proportion than in other regions, whereas standard ICUs were more prevalent in suburban areas, accounting for 59.7% of ICUs (Figure 1). Given the relatively small number of ICUs in rural areas, suburban and rural areas were combined into a single non-metropolitan category for subsequent analyses, and comparisons were conducted between metropolitan and non-metropolitan areas.

### 3.2. ICU Procedure Rate

Procedure rates stratified by ICU admission fee type and geographic category are presented in Table 2. The median procedure rates were 0.81 [0.64–0.91] for AL, 0.27 [0.17–0.40] for CVC, 0.26 [0.17–0.37] for MV, and 0.08 [0.05–0.11] for CRRT. When Poisson regression models were applied to the four procedures, substantial overdispersion was observed, with Pearson χ^2^/df values of 70.9 for AL, 51.8 for CVC, 41.5 for MV, and 30.9 for CRRT. Consequently, quasi-Poisson models were used for the primary analyses. For reference, the Akaike information criterion values of the Poisson models were 47,999 for AL, 32,966 for CVC, 26,859 for MV, and 20,781 for CRRT, and the corresponding residual deviances were 42,338, 28,773, 22,684, and 17,204, respectively. Deviance values were comparable between quasi-Poisson models.

The significance of the interaction between geographic category and ICU admission fee type was evaluated by comparing quasi-Poisson models with and without an interaction term (geographic category × fee type) using F-tests. The interaction was not significant for any procedure (AL, *p* = 0.37; CVC, *p* = 0.054; MV, *p* = 0.28; CRRT, *p* = 0.081); therefore, main-effects models were adopted.

**Figure 2:**
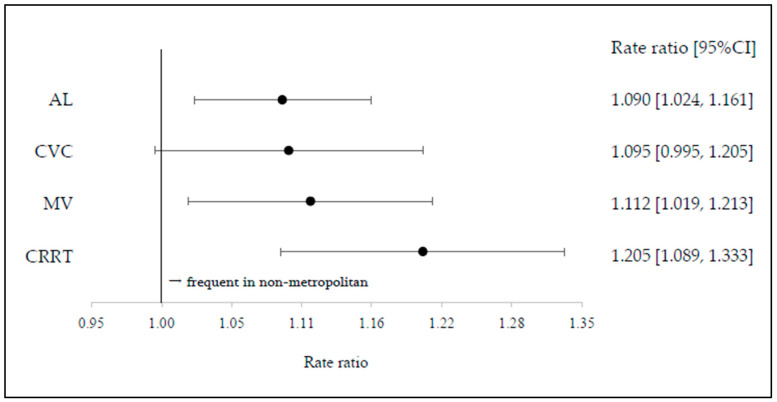
ICU procedure rate ratio by geographic category across metropolitan and non-metropolitan areas. Dots represent rate ratios (point estimates), and horizontal lines indicate 95% confidence intervals.

In the main-effects models, geographic category (metropolitan vs. non-metropolitan) was significantly associated with procedural rates for AL (*p* = 0.006), CVC (*p* = 0.031), MV (*p* = 0.012), and CRRT (*p* = 0.001). ICU admission fee type (high-acuity vs. standard) was significantly associated with procedural rates for AL (*p* < 0.001), CVC (*p* = 0.002), and MV (*p* < 0.001), but not for CRRT (*p* = 0.84). Compared with metropolitan areas, non-metropolitan areas had higher procedural rates for AL (RR 1.090 [95% CI, 1.024–1.161]), MV (RR, 1.112 [1.019–1.213]), and CRRT (RR, 1.205 [1.089–1.333]), whereas the difference for CVC was not significant (RR, 1.095 [0.995–1.205]) (Figure 2).

**Figure 3:**
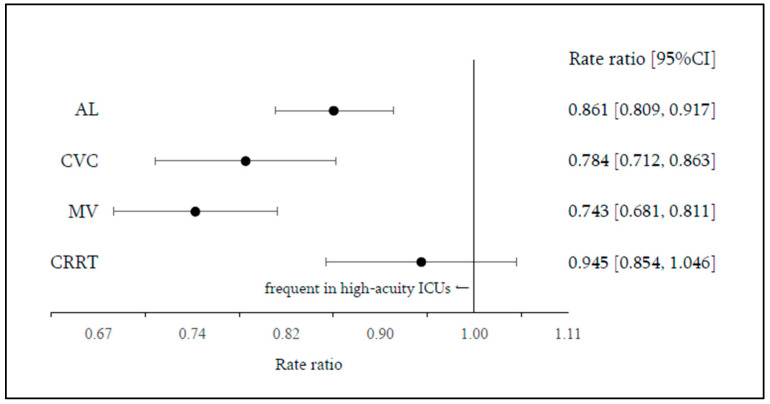
ICU procedure rate ratio by ICU admission fee type: high-acuity ICUs vs. standard ICUs. Dots represent rate ratios (point estimates), and horizontal lines indicate 95% confidence intervals.

When standard ICUs were compared with high-acuity ICUs, procedure rates were lower in standard ICUs for AL (RR, 0.861 [0.809–0.917]), CVC (RR, 0.784 [0.712–0.864]), and MV (RR, 0.743 [0.681–0.811]), whereas no significant difference was observed for CRRT (RR, 0.945 [0.854–1.046]) (Figure 3).

### 3.3. Group Analysis

**Figure 4:**
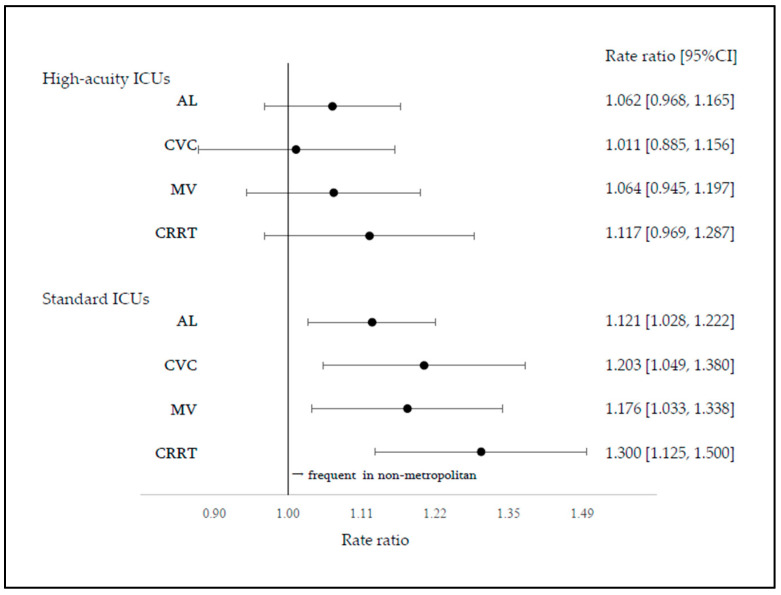
Group analysis: fee type. Dots represent rate ratios (point estimates), and horizontal lines indicate 95% confidence intervals.

**Figure 5:**
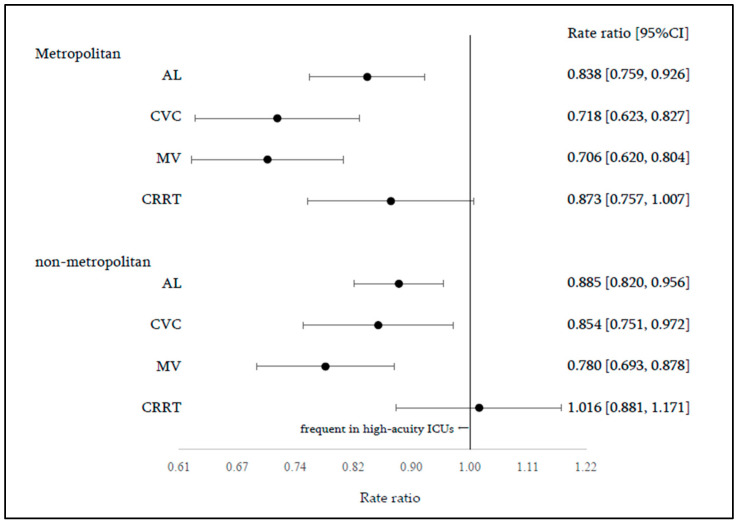
Group analysis: geographic categories. Dots represent rate ratios (point estimates), and horizontal lines indicate 95% confidence intervals.

Regional differences in procedure rates were compared in the group analyses stratified by ICU admission fee type (Figure 4). Among the high-acuity ICUs, no significant differences were observed between the metropolitan and non-metropolitan areas for any procedure (AL, RR = 1.062 [0.968–1.165]; CVC, RR = 1.011 [0.885–1.156]; MV, RR = 1.064 [0.945–1.197]; CRRT, RR = 1.117 [0.969–1.287]). In contrast, among the standard ICUs, procedure rates were consistently higher in non-metropolitan areas for all procedures (AL, RR = 1.121 [1.028–1.222]; CVC, RR = 1.203 [1.049–1.380]; MV, RR = 1.176 [1.033–1.338]; CRRT, RR = 1.300 [1.125–1.500]). Additional analyses stratified by geographic category compared procedure rates between the ICU admission fee types (Figure 5). In metropolitan areas, procedure rates were lower in standard ICUs than in high-acuity ICUs for AL (RR = 0.838 [0.759–0.926]), CVC (RR = 0.718 [0.623–0.827]), and MV (RR = 0.706 [0.620–0.804]), whereas no significant difference was observed for CRRT (RR = 0.873 [0.757–1.007]). Similar trends were observed in non-metropolitan areas (AL, RR = 0.885 [0.820–0.956]; CVC, RR = 0.854 [0.751–0.972]; MV, RR = 0.780 [0.693–0.878]; CRRT, RR = 1.016 [0.881–1.171]).

### 3.4. Sensitivity Analysis

The results of the sensitivity analyses using alternative imputed values (1 or 9) for the masked procedure counts are presented in Table 3. Although greater variability was observed for CRRT, depending on the imputed value, the overall interpretation of the results was generally consistent with that of the primary analysis using an imputed value of 5.

## 4. Discussion

This study demonstrated that nationwide comparisons of procedure frequencies in ICUs are feasible using the Bed Function Report database. Although its ward-level aggregation and claims-based nature impose some limitations, the database comprehensively covers all medical institutions in Japan. Our analyses suggest that, when appropriate research questions are formulated, this open database can be used to visualize structural characteristics of healthcare delivery systems. In practice, this framework provides a scalable, first-step approach for identifying structural patterns in ICU treatment at the national level, thereby facilitating more focused and efficient downstream analyses using patient-level databases.

Notably, high-acuity ICUs were more prevalent in metropolitan areas than in other regions. Nevertheless, procedure frequencies for AL, MV, and CRRT were higher in non-metropolitan areas, whereas procedure rates were consistently lower in standard ICUs than in high-acuity ICUs. Although these differences corresponded to modest effect sizes, they contrasted with the higher concentration of high-acuity ICUs in metropolitan areas. These results indicate that the provision of intensive care is not determined solely by facility classification or geographic category but may also be influenced by structural factors such as operational practices and patient admission patterns.

No significant regional differences in procedure frequencies were observed among high-acuity ICUs, whereas all procedures were performed more frequently in non-metropolitan areas among standard ICUs. These findings should be interpreted cautiously, as the present data did not include information on patient severity or clinical outcomes. Nonetheless, given that ICU admission criteria are not standardized nationwide in Japan, critically ill patients in certain regions are likely receiving treatment in ICUs that do not meet the structural requirements for high-acuity ICUs. Importantly, these findings do not directly reflect differences in the quality of ICU treatment or patient outcomes but rather represent structural patterns of healthcare delivery systems. This study illustrates how the Bed Function Report can be used to capture differences in healthcare structures arising from the interaction between regional context and ICU organizational characteristics.

Previous studies provide context for these observations. Ohbe et al. reported that ICU admission is essential for patients requiring MV in terms of mortality, while also demonstrating that whether such patients are managed in ICUs is influenced more by facility- and region-level factors than by patient characteristics [18,19]. Additionally, ICUs with higher staffing levels are associated with lower ICU and in-hospital mortality [20]. Collectively, these findings suggest that regional differences in ICU structures may contribute to disparities in healthcare delivery in non-metropolitan areas.

Moreover, it should be noted that clinical registries, such as JIPAD, rely on voluntary participation of individual ICUs. Because participation requires continuous data entry and institutional commitment, such registries tend to include larger, resource-rich hospitals and may not fully represent nationwide practice patterns. This voluntary structure introduces inherent selection bias, which may limit the generalizability of registry-based findings. In contrast, the Bed Function Report is mandatorily submitted by all eligible institutions, providing comprehensive national coverage, albeit with reduced clinical granularity. According to the 2023 JIPAD annual report [9], the domestic ICU registry reported procedure frequencies of 93.7% for AL, 47.3% for CVC, 38.1% for MV, and 6.0% for CRRT. As JIPAD participation is more common among facilities with higher admission fee categories and greater staffing resources, these frequencies are likely higher than those observed in the nationwide ICU population. Conversely, because the Bed Function Report is automatically aggregated from claims data, it may underestimate procedure frequencies compared with JIPAD, which relies on manual data entry of performed procedures. These differences highlight the complementary roles of clinical registries and open administrative databases, rather than a direct comparison of superiority.

International comparisons of intensive care systems have highlighted substantial variation in ICU organization, admission practices, and resource allocation across countries, largely reflecting differences in reimbursement structures, regulatory frameworks, and healthcare delivery models. Previous studies have emphasized that cross-national benchmarking requires careful harmonization of definitions and data sources, as structural differences may influence both measured procedure rates and patient outcomes [21]. In several countries, publicly available ICU datasets, such as large electronic health record repositories, have facilitated reproducible research, although their scope and coverage vary considerably across healthcare systems [22]. In this context, although the present findings reflect structural characteristics within the Japanese healthcare system, the methodological framework of using an open nationwide administrative database to profile ICU practice patterns may be adaptable to other settings where comparable public data are available.

Evaluating specific healthcare indicators using existing records continuously registered by all medical institutions enables visualization of structural imbalances in healthcare delivery and provides a practical foundation for policy initiatives aimed at reducing disparities [23]. A stepwise research approach may be advantageous for comprehensive analysis and effective policy development. Nationwide open databases, such as the Bed Function Report, can first be used to identify broad structural patterns, followed by focused analyses using patient-level data sources, such as the DPC database. This study can be positioned as an initial step in such a framework, demonstrating the utility of nationwide open data for profiling structural characteristics of intensive care delivery.

This study had several limitations. First, the Bed Function Report provides ward-level data and does not allow evaluation of individual patient characteristics or clinical severity. Additionally, procedure counts were masked when the number of cases was small. To address this, sensitivity analyses were conducted by imputing masked values using the minimum (1), median (5), and maximum (9) possible values. Although some variability was observed for CRRT, which had a relatively large proportion of masked data, the overall findings for the other procedures were consistent across imputations. These results suggest that the database is suitable for comparative analyses under certain conditions. As masking occurred more frequently for procedures with lower absolute counts, such as CRRT, the potential impact on rate estimation may be greater for low-frequency interventions than for commonly performed procedures.

Second, wards claiming emergency admission fees or pediatric ICU admission fees, which are often considered ICU-equivalent settings, were not included in this study. By restricting the analysis to standard adult ICUs and focusing on four representative procedures, we aimed to demonstrate a generalizable methodological approach for evaluating healthcare delivery structures. Third, this study was based on 2023 data. As revisions to the reimbursement system introduced new ICU admission fee categories in 2024, further evaluation using data collected after these changes will be necessary. Fourth, this study was conducted within a single national healthcare system. Although the inclusion of nearly all ICU wards in Japan enhances internal completeness, the findings may not be directly generalizable to other countries with different reimbursement structures, ICU classifications, and healthcare delivery models. Therefore, caution is warranted when extrapolating these results to other healthcare systems.

## 5. Conclusions

This study demonstrated that nationwide comparisons of ICU practice patterns are feasible using the Bed Function Report. Using ICUs as an example, statistically significant differences in procedure rates were identified according to geographic region and ICU admission fee type, suggesting that structural differences in healthcare delivery can be captured using this database. A stepwise research approach that combines broad profiling with open administrative data, followed by more detailed patient-level analyses, may provide an efficient and practical framework for health services research.

## Figures and Tables

**Table 1:** Characteristics of secondary medical areas and intensive care unit (ICU) distributions in Japan.

	Secondary Medical Area		
	Metropolitan	Suburban	Rural
Population (millions) ^a^	57.5	57.3	11.4
Area (km^2^) ^a^	18,617	180,963	173,379
Population density (/km^2^)	3088.6	315.0	65.8
Number of medical doctors ^a^	116,068	109,844	92,755
Density of medical doctors (/km^2^)	6.23	0.61	0.10
Number of hospitals ^a^	2861	4201	1143
Number of ICUs according to different admission fee types ^b^			
High acuity ICU (*n* = 248)	133	112	3
Standard ICU (*n* = 354)	166	166	22
*p* < 0.01			

^a^ Data were collected from reference [17]. ^b^ Data were collected from reference [3].

**Table 2:** ICU procedure rate stratified by ICU admission fee type or geographic category.

	Median [Interquartile Range]			
	AL	CVC	MV	CRRT
All	0.81 [0.64–0.91]	0.27 [0.17–0.40]	0.26 [0.17–0.37]	0.08 [0.05–0.11]
High acuity ICU	0.87 [0.75–0.93]	0.33 [0.22–0.45]	0.33 [0.22–0.42]	0.08 [0.06–0.11]
Standard ICU	0.76 [0.56–0.87]	0.23 [0.16–0.36]	0.22 [0.15–0.32]	0.08 [0.05–0.12]
Metropolitan	0.80 [0.55–0.90]	0.26 [0.17–0.39]	0.24 [0.15–0.37]	0.07 [0.05–0.11]
non-metropolitan	0.82 [0.70–0.91]	0.28 [0.17–0.41]	0.27 [0.18–0.37]	0.08 [0.06–0.12]

AL, arterial line placement; CRRT, continuous renal replacement therapy; CVC, central venous catheter insertion; MV, mechanical ventilation.

**Table 3:** Sensitivity analyses using alternative imputation values for masked ICU procedure counts.

	Rate Ratio [95% CI]		
	Imputed Value 1	Imputed Value 5	Imputed Value 9
Metropolitan vs. non-metropolitan			
AL	1.093 [1.023–1.167]	1.090 [1.024–1.161]	1.087 [1.024–1.155]
CVC	1.095 [0.973–1.233]	1.095 [0.995–1.205]	1.095 [1.008–1.189]
MV	1.124 [1.005–1.257]	1.112 [1.019–1.213]	1.103 [1.022–1.189]
CRRT	1.395 [1.170–1.664]	1.205 [1.089–1.333]	1.177 [1.066–1.299]
High-acuity ICU vs. standard ICU			
AL	0.854 [0.799–0.912]	0.861 [0.809–0.917]	0.869 [0.818–0.923]
CVC	0.684 [0.607–0.771]	0.784 [0.712–0.863]	0.875 [0.806–0.951]
MV	0.619 [0.552–0.694]	0.743 [0.681–0.811]	0.857 [0.794–0.924]
CRRT	0.626 [0.524–0.748]	0.945 [0.854–1.046]	1.010 [0.915–1.115]
Group analysis			
High-acuity ICU (metropolitan vs. non-metropolitan)			
AL	1.065 [0.968–1.171]	1.062 [0.968–1.165]	1.059 [0.968–1.158]
CVC	1.010 [0.870–1.174]	1.011 [0.885–1.156]	1.012 [0.895–1.145]
MV	1.069 [0.936–1.220]	1.064 [0.945–1.197]	1.059 [0.949–1.182]
CRRT	1.359 [1.018–1.814]	1.117 [0.969–1.287]	1.074 [0.936–1.232]
Standard ICU (metropolitan vs. non-metropolitan)			
AL	1.121 [1.028–1.222]	1.121 [1.028–1.222]	1.118 [1.031–1.213]
CVC	1.203 [1.049–1.380]	1.203 [1.049–1.380]	1.191 [1.064–1.332]
MV	1.176 [1.033–1.338]	1.176 [1.033–1.338]	1.152 [1.037–1.281]
CRRT	1.300 [1.125–1.500]	1.300 [1.125–1.500]	1.281 [1.113–1.475]
Metropolitan (high-acuity ICU vs. standard ICU)			
AL	0.831 [0.748–0.922]	0.838 [0.759–0.926]	0.846 [0.769–0.930]
CVC	0.621 [0.521–0.741]	0.718 [0.623–0.827]	0.807 [0.714–0.912]
MV	0.580 [0.490–0.688]	0.706 [0.620–0.804]	0.821 [0.734–0.918]
CRRT	0.603 [0.484–0.753]	0.873 [0.757–1.007]	0.922 [0.800–1.062]
Non-metropolitan (high-acuity ICU vs. standard ICU)			
AL	0.877 [0.809–0.951]	0.885 [0.820–0.956]	0.893 [0.830–0.961]
CVC	0.751 [0.638–0.884]	0.854 [0.751–0.972]	0.949 [0.850–1.060]
MV	0.658 [0.565–0.767]	0.780 [0.693–0.878]	0.893 [0.806–0.990]
CRRT	0.644 [0.491–0.846]	1.016 [0.881–1.171]	1.100 [0.958–1.263]

AL, arterial line placement; CRRT, continuous renal replacement therapy; CVC, central venous catheter insertion; MV, mechanical ventilation.

## Data Availability

The data supporting the findings of this study are publicly available in the Bed Function Report, provided by the Ministry of Health, Labor and Welfare of Japan, and can be accessed at https://www.mhlw.go.jp/stf/seisakunitsuite/bunya/open_data_00016.html (accessed on 20 May 2025).

## Abbreviations

The following abbreviations are used in this manuscript:

AL	Arterial line placement
CI	Confidence interval
CRRT	Continuous renal replacement therapy
CVC	Central venous catheter insertion
DPC	Diagnosis Procedure Combination
ICU	Intensive care unit
JIPAD	Japanese Intensive Care Patient Database
MV	Mechanical ventilation
PICU	Pediatric intensive care unit
RR	Rate ratio

## References

[1] Beane A., Salluh J.I.F., Haniffa R. (2021). What intensive care registries can teach us about outcomes. Curr. Opin. Crit. Care.

[2] Nascimento Silva P., Nascimento Silva S. (2025). Open government data in the health sector: A systematic literature review. BMC Public Health.

[3] Ministry of Health, Labour and Welfare, Japan Report in the Bed Functions (Open Data). https://www.mhlw.go.jp/stf/seisakunitsuite/bunya/open_data_00016.html.

[4] Ishimaru M., Taira K., Zaitsu T., Inoue Y., Kino S., Takahashi H., Tamiya N. (2022). Characteristics of hospitals employing dentists and utilization of dental care services for hospitalized patients in Japan: A nationwide cross-sectional study. Int. J. Environ. Res. Public Health.

[5] Watanabe H., Miyata S., Kanamori S., Nakata Y. (2024). Health and productivity management associated with improved efficiency of inpatient health care: A cross-sectional study using the fiscal year 2021 bed function report. Inquiry.

[6] Hattori S., Sakata N., Ishimaru M., Tamiya N. (2023). Consolidation of the perinatal care system and workload of obstetricians: An ecological study in Japan. Front. Glob. Womens Health.

[7] Sato S., Yasunaga H. (2023). A review of studies using Japanese nationwide administrative claims databases. Ann. Clin. Epidemiol..

[8] Irie H., Okamoto H., Uchino S., Endo H., Uchida M., Kawasaki T., Kumasawa J., Tagami T., Shigemitsu H., Hashiba E. (2020). The Japanese Intensive Care Patient Database (JIPAD): A national intensive care unit registry in Japan. J. Crit. Care.

[9] Japanese Society of Intensive Care Medicine Japanese Intensive Care Patient Database (JIPAD) Annual Reports. https://www.jipad.org/report.

[10] Masaki H., Suzuki S., Nakayama N., Kobayashi E., Fujii A., Nishiwaki K., Mizuno M., Nakatochi M. (2025). Risk markers for postoperative reintubation of intensive care unit patients: A retrospective multicentre study of the national intensive care registry. Intensive Crit. Care Nurs..

[11] Shiotsuka J., Masuyama T., Uchino S., Sasabuchi Y., Suzuki R., Ono S., Yoshinaga K., Iizuka Y., Sanui M. (2025). Utilization and outcomes of life-supporting interventions in older ICU patients in Japan: A nationwide registry study. Intensive Care Med..

[12] Suzuki Y., Aoki Y., Shimizu M., Nakajima M., Imai R., Okada Y., Mimuro S., Nakajima Y. (2024). Predictive accuracy of the lactate–albumin ratio for mortality in intensive care units: A nationwide cohort study. BMJ Open.

[13] Ohbe H., Sasabuchi Y., Matsui H., Fushimi K., Yasunaga H. (2021). Resource-rich intensive care units versus standard intensive care units on patient mortality: A nationwide inpatient database study. JMA J..

[14] Tanihara S., Kobayashi Y., Une H., Kawachi I. (2011). Urbanization and physician maldistribution: A longitudinal study in Japan. BMC Health Serv. Res..

[15] Kaneko M., Ohta R., Mathews M. (2025). Rural and urban disparities in access and quality of healthcare in the Japanese healthcare system: A scoping review. BMC Health Serv. Res..

[16] Baker O., Horvitz-Lennon M., Yu H. (2024). Clinician distribution and type in rural and urban areas of the National Health Services Corps. JAMA Netw. Open.

[17] Welfare and Medical Service Agency Categorization of Secondary Medical Care Areas into Metropolitan, Regional, and Depopulated Areas. https://www.wam.go.jp/content/wamnet/pcpub/top/fukushiiryokeiei/houjin/houjin001.html.

[18] Ohbe H., Shime N., Yamana H., Goto T., Sasabuchi Y., Kudo D., Matsui H., Yasunaga H., Kushimoto S. (2024). Hospital and regional variations in intensive care unit admission for patients with invasive mechanical ventilation. J. Intensive Care.

[19] Ohbe H., Sasabuchi Y., Yamana H., Matsui H., Yasunaga H. (2021). Intensive care unit versus high-dependency care unit for mechanically ventilated patients with pneumonia: A nationwide comparative effectiveness study. Lancet Reg. Health West Pac..

[20] Ikumi S., Shiga T., Ueda T., Takaya E., Iwasaki Y., Kaiho Y., Tarasawa K., Fushimi K., Ito Y., Fujimori K. (2023). Intensive care unit mortality and cost-effectiveness associated with intensivist staffing: A Japanese nationwide observational study. J. Intensive Care.

[21] Prin M., Wunsch H. (2012). International comparisons of intensive care: Informing outcomes and improving standards. Curr. Opin. Crit. Care.

[22] Jagesar A.R., Dam T.A., Struja T., Sauer C.M., Otten M., Biesheuvel L.A., Girbes A.R.J., Adhikari L., Zhang Z., Faltys M. (2025). Sharing is caring: A systematic review of publicly available intensive care data sets. J. Crit. Care.

[23] Smith M.A., Gigot M., Harburn A., Bednarz L., Curtis K., Mathew J., Farrar-Edwards D. (2023). Insights into measuring health disparities using electronic health records from a statewide network of health systems: A case study. J. Clin. Transl. Sci..

